# The possible occurrence of iron-dependent anaerobic methane oxidation in an Archean Ocean analogue

**DOI:** 10.1038/s41598-021-81210-x

**Published:** 2021-01-15

**Authors:** Fleur A. E. Roland, Alberto V. Borges, François Darchambeau, Marc Llirós, Jean-Pierre Descy, Cédric Morana

**Affiliations:** 1 Chemical Oceanography Unit, Université de Liège, Liège, Belgium; 2 Department of Genetics and Microbiology, Universitat Autònoma de Barcelona, Barcelona, Spain; 3 Department of Earth and Environmental Sciences, Katholieke Universiteit Leuven (KU Leuven), Leuven, Belgium; 4 Present Address: Girona Biomedical Research Institute, Salt, Catalunya Spain

**Keywords:** Biogeochemistry, Limnology

## Abstract

In the ferruginous and anoxic early Earth oceans, photoferrotrophy drove most of the biological production before the advent of oxygenic photosynthesis, but its association with ferric iron (Fe^3+^) dependent anaerobic methane (CH_4_) oxidation (AOM) has been poorly investigated. We studied AOM in Kabuno Bay, a modern analogue to the Archean Ocean (anoxic bottom waters and dissolved Fe concentrations > 600 µmol L^−1^). Aerobic and anaerobic CH_4_ oxidation rates up to 0.12 ± 0.03 and 51 ± 1 µmol L^−1^ d^−1^, respectively, were put in evidence. In the Fe oxidation–reduction zone, we observed high concentration of Bacteriochlorophyll e (biomarker of the anoxygenic photoautotrophs), which co-occurred with the maximum CH_4_ oxidation peaks, and a high abundance of Candidatus Methanoperedens, which can couple AOM to Fe^3+^ reduction. In addition, comparison of measured CH_4_ oxidation rates with electron acceptor fluxes suggest that AOM could mainly rely on Fe^3+^ produced by photoferrotrophs. Further experiments specifically targeted to investigate the interactions between photoferrotrophs and AOM would be of considerable interest. Indeed, ferric Fe^3+^-driven AOM has been poorly envisaged as a possible metabolic process in the Archean ocean, but this can potentially change the conceptualization and modelling of metabolic and geochemical processes controlling climate conditions in the Early Earth.

## Introduction

Tropical inland waters and wetlands have been recognized as major sources of methane (CH_4_) to the atmosphere^1^. While progress has been made in refining the evaluation of the CH_4_ emission rates, less attention has been given to evaluate the underlying production and loss terms, i.e. methanogenesis and methane oxidation. In modern marine sediments where sulfate (SO_4_^2−^) is more abundant by several orders of magnitude than any other electron acceptor, most of the CH_4_ removal is due to anaerobic CH_4_ oxidation (AOM) coupled to SO_4_^2−^ reduction^2–5^. However, SO_4_^2−^ abundance is typically much lower in freshwaters compared to marine ecosystems, so that CH_4_ oxidation in anoxic hypolimnion or sediments of lakes might be linked to the reduction of thermodynamically more favorable electron acceptors such as nitrite (NO_2_^−^)^6^, nitrate (NO_3_^−^)^7^, manganese IV (Mn^4+^) and ferric iron (Fe^3+^)^8^.

Kabuno Bay is a ferruginous, nearly isolated, sub-basin of Lake Kivu (RD Congo) with a marked and distinct physico-chemistry. Primarily due to high hydrothermal activity, a strong and stable stratification is established within Kabuno bay water column throughout the year, with waters being anoxic below ~ 11 m depth^9,10^. A consequence of this strong stratification is the occurrence of a particularly steep gradient in CH_4_ and iron (Fe^2+^/Fe^3+^) concentrations in the chemocline. Also, anoxic waters of Kabuno Bay are characterized by low sulfide (HS^−^) concentrations^11^. These combined features are rarely encountered in modern environments, Lake Matano (Indonesia) and Lake La Cruz (Spain) being one of the few others^12^, while they were widespread in the Archean ocean^13^. Llirós et al.^11^ reported the occurrence of a particularly active pelagic Fe cycle driven by photoferrotrophy in Kabuno Bay, with little net Fe oxidation, meaning that Fe reduction processes are tightly coupled to photoferrotrophic Fe oxidation. In the present study, we measured CH_4_ oxidation rates in the water column of Kabuno Bay, and investigated the potential importance of Fe^3+^ as a terminal electron acceptor for AOM. We hypothesized that Fe^3+^ could be the main electron acceptor for AOM given the high abundance of Fe species in the water column and the high in situ photoferrotrophic rates previously reported in Kabuno Bay^11,14^.

## Material and methods

### Description of the study site and the sampling device

Kabuno Bay (− 1.6216°N, 29.0497°E; Figure S1) was sampled in May 2013 (late rainy season), September 2013 (dry season) and August 2014 (dry season)^15^. Vertical profiles of temperature, conductivity, pH and oxygen were obtained with a Yellow Springs Instrument (YSI) 6600 V2 multiparameter probe, with a detection limit for dissolved oxygen of 0.01 mg L^−1^. High amounts of dissolved gases (in particular CO_2_) were present in superficial and deep waters of Kabuno Bay, causing losses of CH_4_ when samples were brought to the surface. To avoid that, a home-made sampler (Figure S2) was used; sealed N_2_-flushed 60 mL glass serum bottles (SUPELCO, Sigma Aldrich, 33109-U) were fixed on a two-meter high plate, every 0.25 m. Thin needles (0.6 × 25 mm) equipped with non-return valves (valves allowing the water to fill in the bottles but preventing gases to escape from the bottles) penetrated the grey butyl stoppers (WHEATON, USA). The non-return valves were sealed by a butyl stopper. A string was connected to stoppers in series (all stoppers were connected to the same string). The device was immersed at the sampling depth, and the stoppers were removed from the non-return valves by pulling the string, allowing water to enter the bottles through the needle. The system was left under water 10 min to fill the serum bottles. Once the sampling device was brought back to the surface, the needles were removed from the butyl stoppers and further processed as described below. Serum bottles were half-filled with water, and the other half was a N_2_ headspace.

The five main rivers of Kabuno Bay (Figure S1) were sampled every month from November 2013 to June 2014, by the mean of a Niskin bottle. Samples for total Fe and Mn concentrations determination were taken in plastic vials, stored at 4 °C and analyzed as described hereafter.

### Chemical analyses

Samples for CH_4_ concentrations were collected in sealed (with butyl stoppers previously boiled in milli-Q water in the laboratory, and aluminium caps) N_2_-flushed 60 mL glass serum bottles, as described above. Two bottles were directly poisoned with 100 µL of HgCl_2_. CH_4_ concentrations were determined via the headspace equilibration technique and measured by gas chromatography (GC)^16^, as described by Borges et al.^1^. The precision of measurements was ± 3.9% and the detection limit of the method is 0.5 nmol L^−1^.

Samples for nutrient analyses were collected into 250 mL borosilicate bottles, using the same sampling device as for the gas sampling. Water was then collected from the bottles with a 50 mL-syringe, filtered through a 0.22 µm syringe filter (polyethylsulfone), preserved with 200 µL of H_2_SO_4_ 5 N, and stored frozen. Nitrite (NO_2_^−^) and NO_3_^−^ concentrations were measured by spectrophotometry, by the sulfanilamide method^17^ and the vanadium reduction to NO_2_^−^ method^18^, respectively, while ammonia (NH_4_^+^) was determined with the dichloroisocyanurate–salicylate–nitroprussiate colorimetric method^19^. NO_2_^−^ and NH_4_^+^ were quantified on a Thermo Spectronic Genesys 10vis spectrophotometer using a 5-cm light path, and NO_3_^–^ was determined with a Multiskan Ascent Thermo Scientific multi-well plate reader. The detection limits for these methods were 0.03, 0.15 and 0.3 µmol L^−1^ for NO_2_^−^, NO_3_^−^ and NH_4_^+^, respectively. The concentrations for NO_3_^−^ and NO_2_^−^ are reported here as NO_x_ concentrations (NO_3_^−^ + NO_2_^−^). Nutrients concentrations are not available in May 2013, due to a problem during samples preservation.

Samples for SO_4_^2−^ and sulfide (HS^−^) concentrations were collected in N_2_-flushed 60 mL serum bottles, by the same sampling method as described above. Water was rapidly filtered after collection through a 0.22 µm syringe filter, and collected in 5 mL Cryotube vials and 50 mL plastic vials for SO_4_^2−^ and HS^−^, respectively. Samples were preserved with 20 µL of 20% zinc acetate (ZnAc), for SO_4_^2−^ and 200 µL of ZnAc for HS^−^; both samples were then stored frozen. SO_4_^2−^ concentrations were quantified by ion chromatography (Dionex ICS-1500, with an autosampler Dionex AS50, a guard column Dionex AG22 and an analytical column Dionex IonPac AS22) and HS^−^ concentrations were determined with a Thermo Spectronic Genesys 10vis spectrophotometer, using a 5-cm light path, according to the method described by Cline^20^. The detection limits were 0.5 and 0.25 µmol L^−1^ for SO_4_^2−^ and HS^−^, respectively.

Samples for Fe and Mn measurements were collected into sealed N_2_-flushed 60 mL glass serum bottles, with the sampler described above. Water was rapidly transferred from the bottles to the filtration set with a syringe equipped with a tube, and was passed through 25 mm glass fiber filters^15^. Filters were collected in 2 mL Eppendorf vials and preserved with 1 mL of a HNO_3_^−^ 2% solution, while filtrates were collected into four 2 mL Eppendorf vials and preserved with 20 µL of a HNO_3_ 65% solution. Particulate Fe and Mn concentrations were determined from the filters, which were digested with nitric acid in Teflon bombs in a microwave digestion apparatus (Ethos D, Milestone Inc.) and diluted with milli-Q water to a final volume of 50 mL. Dissolved Mn and Fe concentrations were determined from the filtrates, which were diluted with milli-Q water to a final volume of 50 mL. Fe and Mn concentrations were determined by inductively coupled plasma mass spectrometry (ICP-MS) using dynamic reaction cell (DRC) technology (ICP-MS SCIEX ELAN DRC II, PerkinElmer inc.). Analytical accuracy was verified by a certified reference material (BCR 715, Industrial Effluent Wastewater).

### CH_4_ oxidation rate measurements

Samples for CH_4_ oxidation incubations were collected in N_2_-flushed 60 mL glass serum bottles sealed with butyl stoppers previously boiled in milli-Q water in the laboratory, and aluminum caps, using the sampling device described above. CH_4_ oxidation was determined following the methodology described by Roland et al.^21^. Briefly, two bottles were immediately poisoned with 100 µL of HgCl_2_ after collection (T_0_), five bottles received an inhibitor of sulfate-reducing bacteria (sodium molybdate, + Mo) and five other did not receive any amendment (− Mo). In May 2013, the molybdate (Mo) solution was prepared directly on the field with milli-Q water stored at ambient temperature and was not flushed, while it was prepared with fresh milli-Q water and was flushed before the transport in September 2013 and August 2014. The bottles of both treatments were incubated in the dark and at constant temperature close to in situ temperature (~ 23 °C). The biological activity in + Mo and – Mo bottles was stopped at ~ 12, 24, 48, 72 and 96 h by the addition of 100 µL of HgCl_2_. CH_4_ concentrations were determined via the headspace equilibration technique and measured by gas chromatography (GC)^16^, as described by Borges et al.^1^. The precision of measurements was ± 3.9% and the detection limit of the method is 0.5 nmol L^−1^. The precision was calculated based on the analysis of the two T_0_ bottles sampled in duplicate for each depth and then accounted for the variability induced by the handling of samples (samples collection, storage) and our analytical method.

CH_4_ oxidation rates were calculated as a linear regression of CH_4_ concentrations over time during the course of the incubation. Table S1 shows standard deviations, initial CH_4_ concentrations, percentage of CH_4_ consumed and the time lapse during which the CH_4_ oxidation rates were calculated for each depth^15^.

A correction of the CH_4_ oxidation rates has been applied taking into account the potential oxygen supply through the injection of the Mo solution. We considered that a maximum of 2.5 µmol L^−1^ of O_2_ were added to each bottle (250 µL of the solution were added to 30 mL of water). The calculations were made according to Roland et al.^21^. Only the time change in dissolved CH_4_ concentration and not the concomitant decrease in the concentration of the electron acceptors potentially involved in CH_4_ oxidation processes were monitored during the incubation. This is due to the interference caused by the HgCl_2_ poison addition with the analytical methods used to determine the electron acceptors concentrations.

### Vertical flux calculations

The vertical fluxes (F_vertical_) of NH_4_^+^, SO_4_^2−^, HS^−^, Mn^2+^ and Fe^2+^ were calculated as described by Pasche et al.^22^ (Eq. 1):

1 $$ {\text{F}}_{\text{vertical}} = \, - {\text{ D}}_{\text{turbulent}} *{\text{Grad }} + {\text{ C}}*{\text{Adv}} $$

where D_turbulent_ is the turbulent diffusion coefficient, Grad is the vertical concentration gradient of each element, C is the concentration of the element at a given depth, and Adv is the upwelling velocity. Vertical fluxes were computed by using a range of turbulent diffusion coefficient and of upwelling velocity of 1.4 × 10^–7^–1.0 × 10^–6^ m^2^ s^−1^ and 6.3 × 10^–9^–6.3 × 10^–8^ m s^−1^, respectively^14^.

### Contribution to CH_4_ oxidation

Based on these vertical fluxes, the fraction of the integrated AOM rates potentially sustained by Fe reduction, NO_3_^−^ reduction and SO_4_^2−^ reduction rates were calculated considering stoichiometry equivalences of 1:1 (SO_4_^2−^:CH_4_), 8:5 (NO_3_^−^:CH_4_), 8:1 (Fe(OH)_3_:CH_4_) and 4:1 (MnO_2_:CH_4_)^8,23^, according to the following equations:

2 $$ \begin{aligned} \% {\text{SO}}_{{4}}^{{{2} - }} & = {\text{ vertical SO}}_{{4}}^{{{2} - }} {\text{flux observed/vertical SO}}_{{4}}^{{{2} - }} {\text{flux needed}}*{1}00 \\ & = {\text{ vertical SO}}_{{4}}^{{{2} - }} {\text{flux observed/integrated AOM rates}}*{1}00 \\ \end{aligned} $$

3 $$ \begin{aligned} \% {\text{NO}}_{{3}}^{ - } & = {\text{ vertical NH}}_{{4}}^{ + } {\text{flux observed/vertical NH}}_{{4}}^{ + } {\text{flux needed}}*{1}00 \\ & = {\text{ vertical NH}}_{{4}}^{ + } {\text{flux observed/}}\left( {{\text{integrated AOM rates}}/\left( {{5}*{8}} \right)} \right)*{1}00 \\ \end{aligned} $$

4 $$ \begin{aligned} \% {\text{Fe}}\left( {{\text{OH}}} \right)_{{3}} & = {\text{ vertical Fe}}^{{{2} + }} {\text{flux observed/vertical Fe}}^{{{2} + }} \,{\text{flux needed}}*{1}00 \\ & = {\text{vertical Fe}}^{{{2} + }} {\text{flux observed/}}\left( {{\text{integrated AOM rates}}*{8}} \right)*{1}00 \\ \end{aligned} $$

5 $$ \begin{aligned} \% {\text{MnO}}_{{2}} & = {\text{ vertical Mn}}^{{{2} + }} {\text{flux observed/vertical Mn}}^{{{2} + }} \,{\text{flux needed}}*{1}00 \\ & = {\text{ vertical Mn}}^{{{2} + }} {\text{flux observed/}}\left( {{\text{integrated AOM rates}}*{4}} \right)*{1}00. \\ \end{aligned} $$

### Bacteriochlorophyll pigments analyses

Samples for pigments analyses were collected every 0.25 m, from 9 to 13 m depth in September 2013, and from 8 to 12 m depth in August 2014. Water was collected with the sampler described above, and filtered through Whatman GF/F 47 mm diameter filters. The filtration volume depended on the depth sampled, but was on average 0.3 L. Filters were preserved in 5 mL Cryotube vials and stored frozen. The pigment extraction was made in 4 mL of 90% HPLC grade acetone. Two 15-min sonication steps separated by an overnight period at 4 °C in dark were applied, and extracts were stored in 2 mL-amber borosilicate vials. HPLC analyses were carried out as described by Sarmento et al.^24^.

### Archaeal diversity

Samples for DNA analyses were collected as detailed by Inceoğlu et al.^25^. Genomic DNA was extracted as previously described^26^ and further subjected to FLX–titanium amplicon pyrosequencing^27^ from collected filters using ARCH 349F (5′-GYGCASCAGKCGMGAAW-3′) and ARCH 806R (5′-GGACTACVSGGGTATCTAAT-3′) as sequencing primers targeting the 16S rRNA V3–V4 region^27^. Reads used in the present study can be accessed through sequencing read archive (SRX349388). Archaeal 454-pyrosequencing sequences from Kabuno Bay water samples tentatively belonging to the *Methanosarcinales* order were subsequently analysed for further taxonomic refinement. All sequences were aligned using the SINA aligner^28^ and then imported into the latest SILVA 16S rRNA-ARB-compatible database (SSURef-132_NR_99_13_12_17_opt.arb; http://www.arb-silva.de) in ARB^29^. Two base frequency filters (“termini” and “ssuref:archaea”; positional variability by parsimony) were applied to exclude highly variable positions before adding sequences to the original database using the “parsimony quick add marked” tool from ARB.

## Results and discussion

### Environmental settings in Kabuno Bay

**Figure 1:**
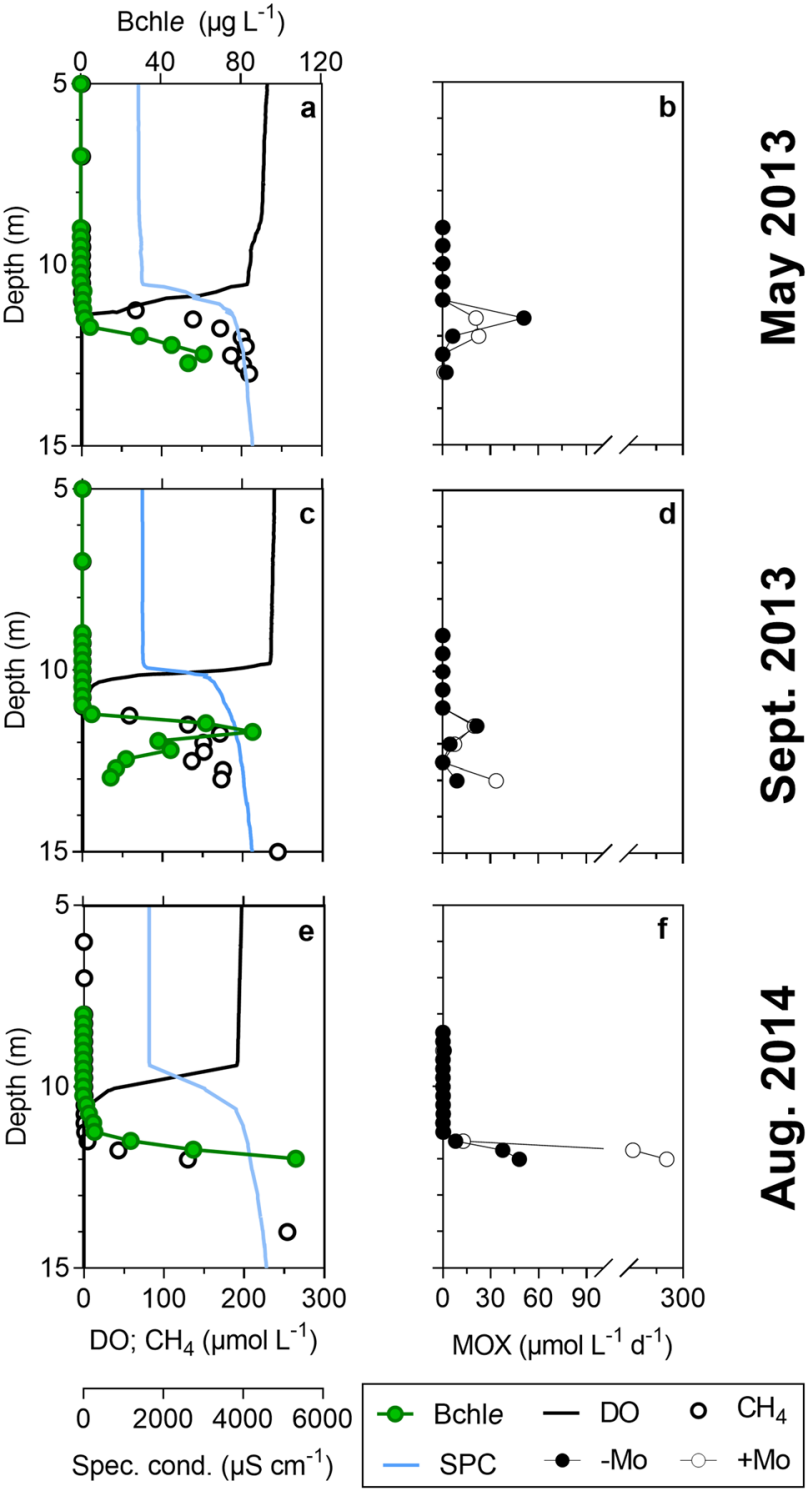
Physico-chemical conditions in Kabuno Bay are analogous to the Archean Ocean (**a**,**b**: May 2013; **c**,**d**: September 2013; **e**,**f**: August 2014). (**a**,**c**,**e**): bacteriochlorophyll contents (Bchl*e*, µg L^−1^), dissolved oxygen (DO, µmol L^−1^) and CH_4_ concentrations (µmol L^−1^), specific conductivity (SPC, µS cm^−1^). (**b**,**d**,**f**): methane oxidation rates (µmol L^−1^ d^−1^) without molybdate added (− Mo) and with molybdate added (+ Mo).

**Figure 2:**
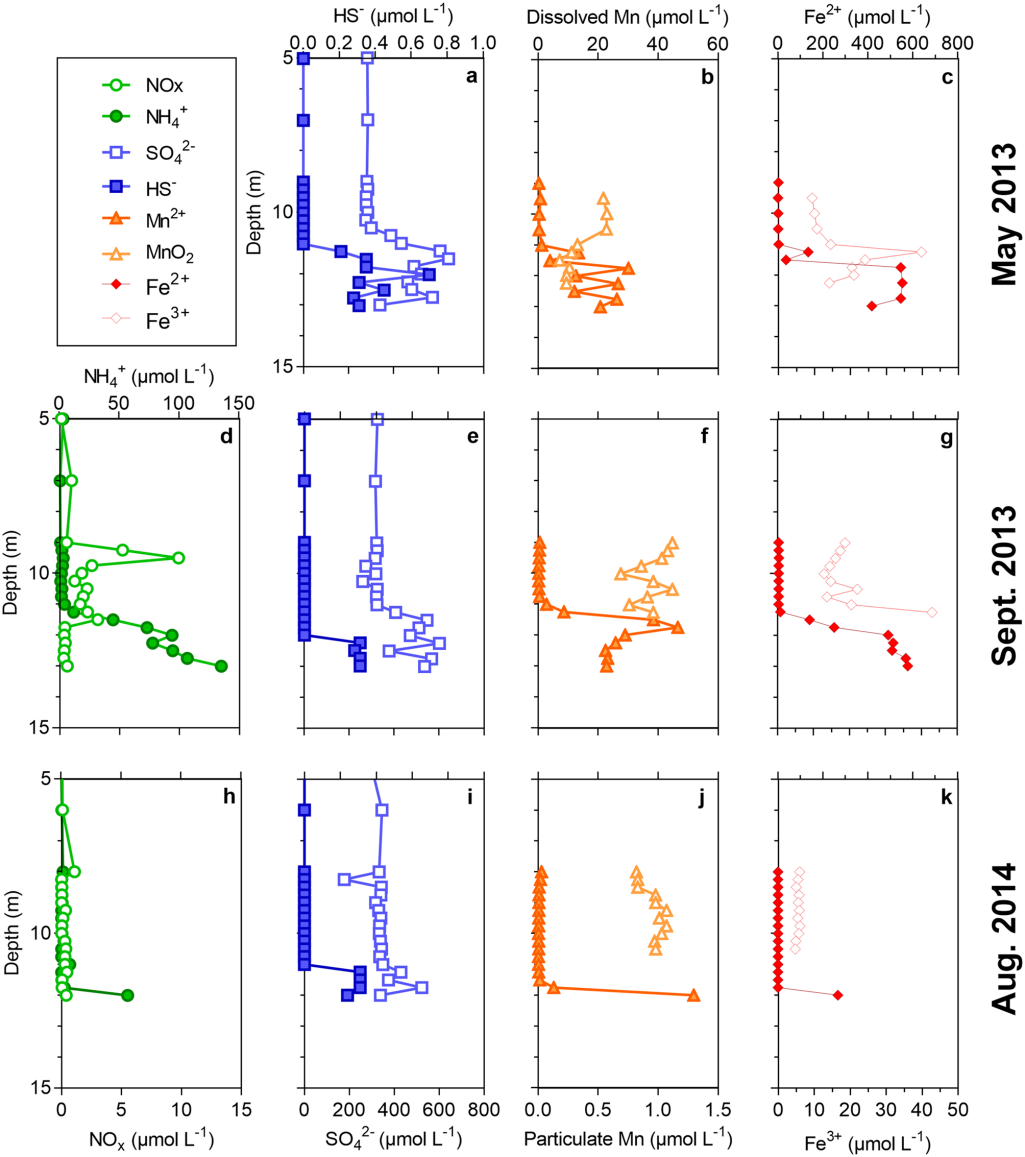
Vertical profiles of electron acceptors potentially involved in AOM (**a**–**c**: May 2013; **d**–**g**: September 2013; **h**–**k**: August 2014). (**d**,**h**): NOx and NH_4_^+^ concentrations (µmol L^−1^). (**a**,**e**,**i**): SO_4_^2−^ and HS^−^ concentrations (µmol L^−1^). (**b**,**f**,**j**(: particulate (MnO_2_) and dissolved (Mn^2+^) Mn concentrations (µmol L^−1^). (**c**,**g**,**k**(: particulate (Fe^3+^) and dissolved (Fe^2+^) Fe concentrations (µmol L^−1^).

The water column of Kabuno Bay was sharply stratified and anoxic from 11.0 to 11.3 m depth during the three field campaigns (2013–2014; Fig. 1, Figure S3). Chemocline co-occurred with the oxycline. Methane was abundant in anoxic waters (up to ~ 200 µmol L^−1^ at 13 m) but its concentration decreased abruptly at the bottom of the chemocline to relatively modest values (0.1–1.1 µmol L^−1^), indicative of vigorous microbial CH_4_ oxidation. Ferruginous Kabuno Bay hypolimnion was characterized by high Mn^2+^ and Fe^2+^ concentrations (up to 55 and 600 µmol L^−1^, respectively; Figs. 1, 2). Dissolved Mn and Fe concentrations declined in the CH_4_ gradient, and were mirrored by an accumulation of particulate Mn and Fe species.

Sulfate concentrations were relatively high in the chemocline and did not change substantially within the CH_4_ gradient, while HS^−^ concentrations were always two orders of magnitude lower than SO_4_^2−^ (i.e., lower than 1.0 µmol L^−1^), with the exception of a maximum peak (10 µmol L^−1^) detected at 9.5 m depth in September 2013 (Fig. 2). The vertical fluxes of the potential electron acceptors for AOM and their reduced forms are shown in Table S2.

Five main rivers enter Kabuno Bay and are sources of Fe and Mn to the lake. Based on data gathered during a monthly monitoring of these rivers, we estimated that they supplied the lake with 3.3 mmol m^−2^ d^−1^ of Fe^3+^ oxide and 0.1 mmol m^−2^ d^−1^ of Mn^4+^ oxide.

### Electron acceptors sustaining AOM in Kabuno Bay

Methane oxidation rates in oxic waters (maximum of 0.12 ± 0.03 µmol L^−1^ d^−1^ observed in May 2013) were one order of magnitude lower than those in anoxic waters with maximum rates of 51 ± 1 (at 11.5 m depth), 21 ± 4 (at 11.5 m) and 48 ± 7 (at 12.0 m) µmol L^−1^ d^−1^ in May 2013, September 2013 and August 2014, respectively (Fig. 1). Methane removal in anoxic waters by aerobic organisms has been found to be supported by oxygenic photosynthesis in the well-illuminated (10% of incident PAR) chemocline of Lake Cadagno^30^. Kabuno Bay chemocline is located below the photic zone so that light conditions (0.1–1% PAR) do not appear suitable to support significant phytoplankton activity. Pigments analysis carried out during our study revealed that the abundance of bacteriochlorophyll *e* (Bchl *e*)*,* a pigment distinctive of low light adapted anoxygenic photoautotrophs Green Sulfur Bacteria (GSB)^31,32^, was at least one order of magnitude higher than chlorophyll *a* in the chemocline (Figs. 1 and S4). Also, Morana et al.^14^ showed that 74 ± 13% of particulate biomass in the chemocline derive from anoxygenic CO_2_ fixation by GSB. These multiple lines of evidence indicate that biological primary production in the chemocline is largely dominated by anoxygenic photoautotrophs so that a cryptic oxygen cycle sustained by oxygenic photosynthesis in Kabuno Bay anaerobic waters can be ruled out. Nevertheless, we cannot rule out the possibility of punctual and/or episodic oxygen incursions in the anoxic waters, which could explain the presence of NO_x_ below the oxycline in September 2013. This oxygen could be rapidly used by aerobic CH_4_ oxidation, and the part of aerobic CH_4_ oxidation in the water column of Kabuno Bay might be more significant than reflected by the results of our in vitro incubations.

However, in the particular conditions of our in vitro incubations, we could estimate that 89–98% of the CH_4_ was oxidized under anoxic conditions, raising the question which electron acceptors supported AOM. The water column was particularly rich in SO_4_^2−^, but SO_4_^2−^ concentrations did not decrease substantially with depth in the chemocline (Fig. 2), neither in anoxic waters. Indeed, SO_4_^2−^ reduction rates reported by Llirós et al.^11^ were relatively low compared to SO_4_^2−^ concentrations and were *ca.* 10 times lower than the AOM rates. Furthermore, except in May 2013 where AOM rates were lower with Mo added (what can be linked to the slightly different methodology used), our in situ incubation experiments revealed that AOM rates were up to 6 times higher (September 2013 and August 2014) in presence of Mo, an inhibitor of SO_4_^2−^ reduction (Fig. 1), contrary to what would have been expected if CH_4_ oxidation depended on SO_4_^2−^ reduction. All these multiple lines of evidence suggest that SO_4_^2−^ reduction did not sustain a significant part of the CH_4_ oxidation in the chemocline of Kabuno Bay. Similarly, it seems unlikely that Mn oxides and NO_3_^−^ would fuel a substantial part of AOM. Their concentrations were always low, and the upward fluxes of Mn^2+^ and NH_4_^+^ could only have sustained at most 4% and 25% of the AOM rates, considering an extreme scenario where the totality of Mn^2+^ and NH_4_^+^ fluxes, once oxidized, would be exclusively involved in Mn oxides or NO_3_^–^dependent AOM, which is unlikely. Instead, it has been showed that denitrification and NO_3_^−^ reduction to NH_4_^+^ (DNRA) linked to Fe oxidation occur in the water column of Kabuno Bay^33^. As both these processes are thermodynamically more favorable than NO_3_^–^ driven AOM, we hypothesized that the NO_3_^−^ formed in the water column of Kabuno Bay is rapidly consumed by DNRA and denitrification coupled to Fe oxidation rather than by NO_3_^–^driven AOM, which can also explain the low NO_3_^−^ concentrations. Therefore, it is likely that AOM coupled to NO_3_^−^ reduction did not occur at a significant extent in Kabuno Bay.

**Figure 3:**
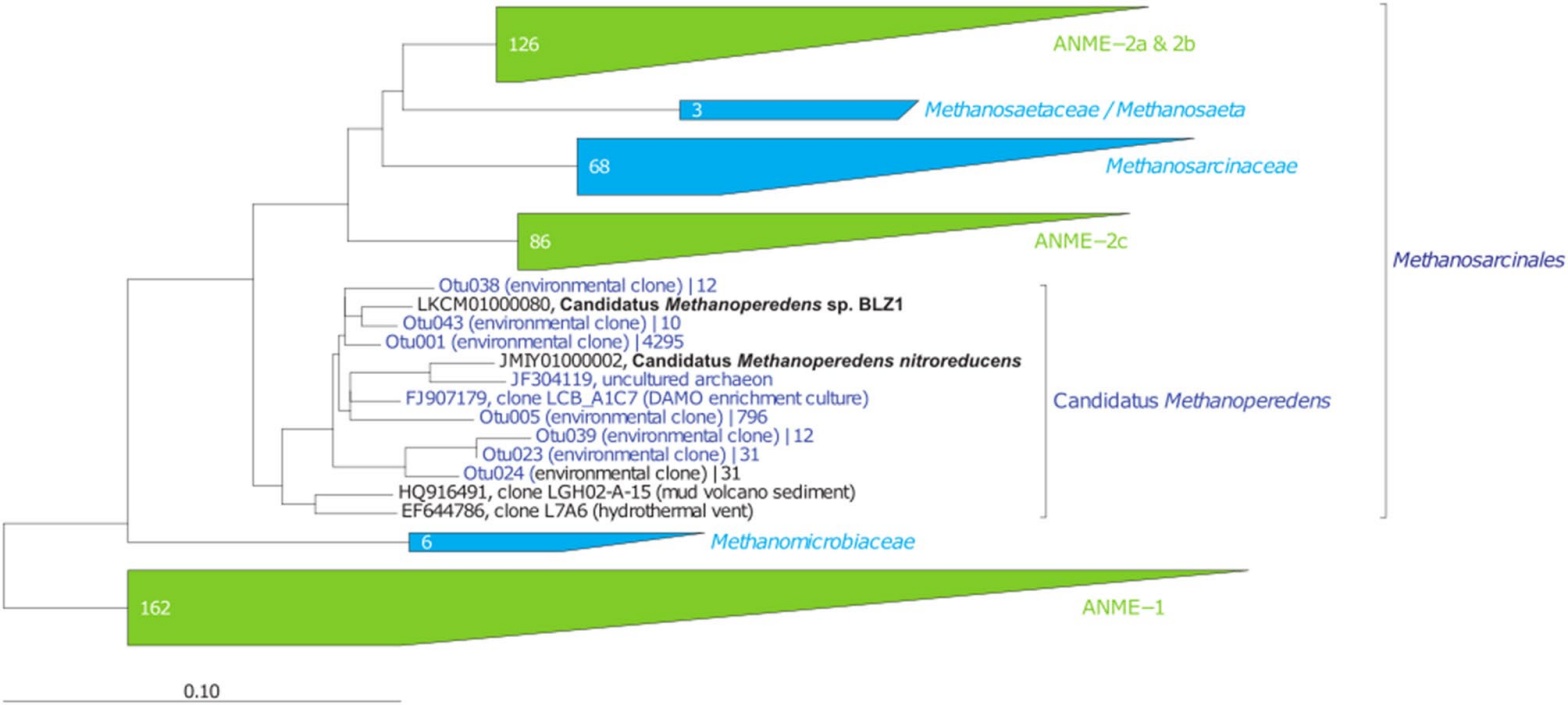
Presence of *Candidatus Methanoperedens*, capable of Fe-related AOM. 16S rRNA gene phylogenetic tree of the *Candidatus Methanoperedens* representative related OTUs (0.03 cut-off; only those OTUs containing more than 10 reads are shown) retrieved by pyrosequencing from Kabuno Bay water samples, the scale bar indicates 0.10 fixed point mutation per nucleotide position.

**Figure 4:**
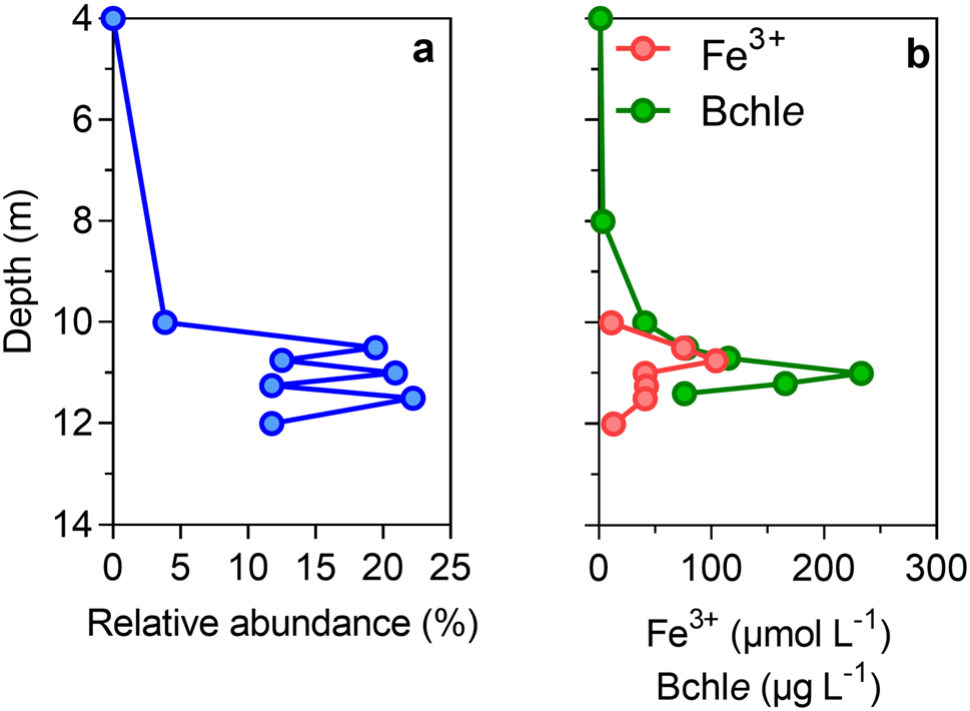
Co-occurence of *Candidatus methanoperedens* and ferrophototrophs in the chemocline of Kabuno Bay. (**a**) Vertical distribution of *Candidatus* methanoperedens and (**b**), Vertical profiles of particulate Fe concentrations (µmol L^−1^) and Bacteriochlorophyll e (Bchl*e*) content (µg L^−1^) in February 2012. While this profile was not contemporary to the measurements of AOM (Fig. 1) the particulate Fe and Bacteriochlorophyll e peaks show that AOM and Archaea abundance coincided.

On the other hand, Llirós et al.^11^ showed that Fe^3+^ reduction in Kabuno Bay was *ca.* 24 times higher than SO_4_^2−^ reduction. This study also reported the existence of an important Fe-related bacterial community in the water column of Kabuno Bay, among which *Chlorobium phaeoferrooxidans*, a GSB capable of Fe oxidation^34^ was the dominant member. Here, we showed that Bchl *e*, a specific biomarker of the GSB, was mainly located in the Fe oxidation–reduction zone, which co-occurred with the maximum CH_4_ oxidation peaks (Fig. 1). 16S-RNA gene based pyrosequencing data from February 2012 showed the presence of an archaeal community related to *Candidatus Methanoperedens nitroreducens* (the most abundant retrieved OTUs showed 94.6 and 93.7% sequence similarity, respectively, against *Candidatus Methanoperedens nitroreducens* (JMIY01000002); Fig. 3 and Table S3). The peaks of abundance of these archaea co-occurred within the Fe oxidation/reduction zone and the maximum occurrence of photoferrotrophy previously reported by Llirós et al.^11^ (Fig. 4). Overall, putative AOM-related archaea in Kabuno bay represented *ca.* 16% of the whole community in February 2012. Recent studies have shown that microbes belonging to the *Candidatus Methanoperedens* archaeal group (Order *Methanosarcinales*) are particularly versatile, and can couple AOM with different electron acceptors, among which Fe, depending on environmental conditions^35,36^. Cai et al. recently demonstrated that these archaea were capable to catalyze Fe-linked AOM alone, without a bacterial partner, via the “reverse methanogenesis” pathway and possibly using a extracellular electron transport pathway^37^. Despite that these prokaryotes diversity data were acquired during a different sampling campaign^25^ than the AOM measurement reported here, the good agreement between (1) high Candidatus methanoperedens abundance and high Bchl *e* concentrations in the Fe oxidation–reduction zone in 2012, and (2) high AOM rates and high Bchl *e* concentrations in the Fe oxidation–reduction zone during this study, allow to hypothesize that Candidatus *methanoperedens* could represent the dominant microbes thriving AOM during the present study.

**Figure 5:**
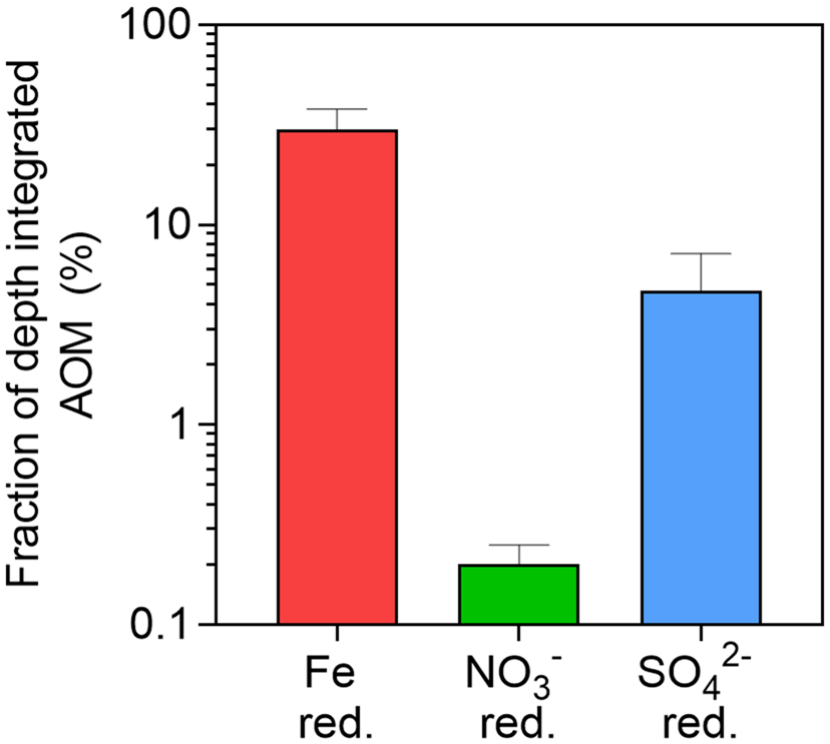
Chemical evidence of Fe^3+^ as an important electron acceptor of AOM. Fraction (%) of the integrated AOM rates potentially sustained by Fe reduction, NO_3_^−^ reduction and SO_4_^2−^ reduction rates measured by Llirós et al.^11^ and Michiels et al.^33^. Error bars are calculated as the standard deviation of the mean of the three sampling campaigns.

Accordingly, these AOM-associated archaea (AAA^5^) could therefore be responsible for the Fe-related AOM measured in Kabuno bay, taking into account that AOM coupled to Fe reduction is thermodynamically more favorable than coupling with other electron acceptors^38^. Iron-driven AOM has already been suggested as a dominant CH_4_ removal pathway in other ferruginous environments (i.e.^38–40^). Bacterial data reported from samples collected in 2012 evidenced that photoferrotrophs were responsible for the oxidation of 37 mmol Fe m^−2^ d^−1^ ^11^. Comparison of this Fe oxidation rate with AOM showed that Fe^3+^ released via photoferrotrophy could potentially fuel an important fraction of the AOM (up to 31% in September 2013), in contrast to NO_3_^−^ and SO_4_^2−^ reduction rates^11,33^, which could only fuel up to 5% of the AOM observed (Fig. 5). Furthermore, the observation of higher AOM rates when Mo was added may result from the higher availability of Fe oxides when sulfate-reducing bacteria (SRB) activity was inhibited. Indeed, SO_4_^2−^ reduction produces HS^−^ that can rapidly reduce Fe oxides to form iron sulfides, which could precipitate and then be unavailable for Fe-dependent CH_4_ oxidizers. Fe is known to be efficiently recycled in the chemocline of ferruginous lakes^38^ or bioturbated marine sediments^41^.

Overall, process rate measurements and metagenomics data suggest that an intense biotic regeneration of Fe^3+^ mediated by photoferrotrophs could provide an important fraction of the electron acceptors required to oxidize CH_4_ anaerobically in Kabuno Bay. Following the same approach as Jones et al.^42^ and assuming steady state conditions, the rate of Fe or Mn leaving the water column via sedimentation must equals the rate of Fe or Mn input in Kabuno bay’s water column via the rivers. Assuming that Mn and Fe leave the water column as a Mn^2+^ or Fe^2+^ mineral, all the oxidized Mn or Fe must ultimately be reduced, and the downward flux of particulate Fe^3+^ should be equivalent to the input of Fe via the rivers (3 mmol m^−2^ d^−1^) and the upward flux of Fe^2+^ (4–33 mmol m^−2^ d^−1^). If we consider that the totality of this downward flux of particulate Fe^3+^ is reduced within Kabuno bay’s chemocline, but that only 3 mmol Fe m^−2^ d^−1^ finally leave the mixolimnion, we estimated that Fe would be recycled up to 11 times before removal by sedimentation.

### Consequences on representation of the Archean ocean metabolism

A strong interaction between photoferrotrophs and CH_4_ oxidizers has been hypothesized in several other modern Archean ocean analogues, such as Lake Matano^38^ and Lake La Cruz^43^. Under the ferruginous conditions of Archean oceans, photoferrotrophs would have been responsible for most of the primary production of the primitive Earth^12^. It is also generally assumed that a much larger fraction of the organic matter generated by primary producers would have been processed by methanogens than nowadays, given the absence of oxic and SO_4_^2−^ driven mineralization of organic matter in the O_2_ and SO_4_^2−^ depleted waters of the early Earth Ocean^44,45^.

Pavlov et al.^46^ estimated that up to 250 Tmol year^−1^ (1.9 mmol m^−2^ d^−1^, assuming an ocean area of 3.6 × 10^14^ m^2^) of CH_4_ would have been emitted during the Archean, hence CH_4_ would have been a key component of the ancient C cycle, with important consequences on the early Earth climate, as the higher CH_4_ concentrations (~ 100 ppmv) in the atmosphere are thought to have provided enough greenhouse warming to compensate for a 5–17% fainter Sun. The photochemical decomposition of CH_4_ in the atmosphere and the resulting escape of hydrogen to space may also have participated to the oxidation of the Earth surface environment^47^. However, geological archives do not provide constraints on the magnitude of the CH_4_ concentrations, and large CH_4_ fluxes are calculated with quantitative models that limit the existence of Fe-dependent CH_4_ oxidation and assume a negligible role of SO_4_^2−^ driven CH_4_ oxidizers. This paradigm has recently been challenged by Olson et al.^48^ and Sauterey et al.^49^ who showed that the combined effects of competition between methanogens and SO_4_^2−^ reducers and occurrence of SO_4_^2−^ driven CH_4_ oxidation would have effectively reduced the CH_4_ fluxes from the ocean even at modest SO_4_^2−^ concentrations, and regardless of O_2_ concentrations. Similarly, we propose that strong interactions between photoferrotrophs and Fe-dependent CH_4_ oxidizers might have exerted an important control on the Archean–Proterozoic CH_4_ cycling. For instance, modelled photoferrotrophic primary production in early Earth ferruginous ocean (3.8 mmol m^−2^ d^−1^ ^50^) would have produced Fe oxides at a rate of 15.3 mmol m^−2^ d^−1^, assuming a 4:1 ratio between Fe oxidation and C fixation by photoferrotrophs. Although simplistic given the complexity of the C and Fe biogeochemical cycle, comparison of this rate of Fe oxides production with the sea-to-air CH_4_ flux (1.9 mmol m^−2^ d^−1^) proposed by Pavlov et al.^46^ suggests that photoferrotrophy would have been high enough to potentially support the oxidation of 100% of the CH_4_ flux to the early atmosphere. In Kabuno Bay, a modern analog to early Earth ocean, measured photoferrotrophy rates would have been sufficient to sustain a smaller (31 ± 18%, *n* = 3), but still important, part of the oxidation of the CH_4_ flux measured during this study (19 ± 11 mmol m^−2^ d^−1^, *n* = 3).

It has been hypothesized that the rise of atmospheric oxygen about 2.4 Gyr ago (Great Oxidation Event) was triggered by a decrease of atmospheric CH_4_ levels^51^. This has been attributed to an increased importance of SO_4_^2^ reduction in the oceans that outcompeted the methanogenic organisms, although this explanation is not consistent with geological records^52^. It has also been hypothesized that the production of methane decreased in response to the decrease of the nickel inputs to oceans, as this is a key metal for methanogen enzymes^53^. Alternatively, it was showed that supplying a culture media with nickel does not stimulate methanogenesis^54^. A widespread occurrence of AOM coupled to Fe oxidation offers an alternative explanation that would have led to the decrease of dissolved CH_4_ in the ocean and consequently the emission of CH_4_ to the atmosphere, thus going in the direction of the study of Riedinger et al.^39^. This is consistent with the revision of the putative composition of the Archean atmosphere^55^ that suggests that the amount of greenhouse warming by CH_4_ was more limited than previously thought^49,56^.

**Figure 6:**
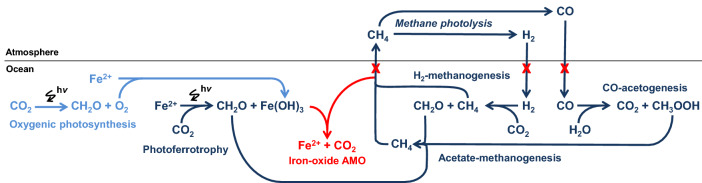
Potential role of iron-oxide AOM in Archean Ocean metabolism. In the Archean Ocean, anaerobic metabolism (photoferrotrophy, methanogenesis and CO-acetogenesis in dark blue) dominated before the advent of oxygenic photosynthesis (in light blue). In red, we propose anaerobic methane oxidation (AOM) using Fe oxides as electron acceptor as additional metabolic process. Before the advent of oxygenic photosynthesis, Fe^3+^ dependent AOM linked photoferrotrophy and H_2_-methanogenesis that so far have been seen as two parallel and unconnected processes. After the advent of oxygenic photosynthesis, Fe oxides-dependent AOM might have used the abundantly produced Fe(OH)_3_ from the oxidation of Fe^2+^ by O_2_ to further remove CH_4_ from the water column, facilitating the increase of O_2_ in the atmosphere and the great oxidation event.

While we can hypothesize that Fe(OH)_3_ sustaining AOM was initially provided by photoferrotrophy, the onset of oxygenic photosynthesis would also have been an ample supply of Fe(OH)_3_, available to sustain AOM, from the oxidation of Fe^2+^ with dissolved O_2_ released by oxygenic photoautotrophs (Fig. 6). This might also provide an explanation to the modest but significant increase in Fe-using genes from the Archean to the Proterozoic given by the phylogenomic analyses^57^. A decrease of dissolved CH_4_ concentrations due to Fe-dependent AOM could have had several other consequences on the timing and sequence of Archean and Proterozoic events. The decrease of atmospheric CH_4_ would have led to a decrease of H_2_ and CO generation by photolysis, which in turn would have led to a decrease of supply of H_2_ and CO to surface oceans, contributing to the demise of pelagic H_2_-methanogens and CO-acetogens (Fig. 6), that is usually attributed exclusively to the inhibition of these anaerobic organisms by increasing O_2_ level.

Finally, the great oxygenation event was shortly afterwards followed by a low latitude glaciation that would be associated to lower atmospheric CH_4_ concentrations. However, the trigger of the collapse of CH_4_ has not been clearly identified, some authors arguing that CH_4_ decreased as a consequence of rising O_2_^58^, while others proposed that low CH_4_ level preceded the transition to an O_2_-richer atmosphere^49,51^. Co-occurrence of photoferrotrophy and Fe-dependent CH_4_ oxidation in the Archean would support the latter hypothesis and the view of Olson et al.^48^ who recently proposed an alternative mechanism for the initiation of low-latitude glaciation with low baseline atmospheric CH_4_ levels.

## Supplementary Information

Supplementary Information.

## References

[1] Borges AV (2015). Globally significant greenhouse-gas emissions from African inland waters. Nat. Geosci..

[2] Iversen N, Jørgensen B (1985). Anaerobic methane oxidation rates at the sulfate-methane transition in marine sediments from Kattegat and Skagerrak (Denmark). Limnol. Oceanogr..

[3] Boetius A (2000). A marine microbial consortium apparently mediating anaerobic oxidation of methane. Nature.

[4] Jørgensen BB, Weber A, Zopfi J (2001). Sulfate reduction and anaerobic methane oxidation in Black Sea sediments. Deep-Sea Res. Pt..

[5] Knittel K, Boetius A (2009). Anaerobic oxidation of methane: progress with an unknown process. Annu. Rev. Microbiol..

[6] Ettwig KF (2010). Nitrite-driven anaerobic methane oxidation by oxygenic bacteria. Nature.

[7] Haroon MF (2013). Anaerobic oxidation of methane coupled to nitrate reduction in a novel archaeal lineage. Nature.

[8] Beal EJ, House CH, Orphan VJ (2009). Manganese-and iron-dependent marine methane oxidation. Science.

[9] Borges AV, Abril G, Delille B, Descy JP, Darchambeau F (2011). Diffusive methane emissions to the atmosphere from Lake Kivu (Eastern Africa). J. Geophys. Res. Biogeosci..

[10] Ross KA, Gashugi E, Gafasi A, Wüest A, Schmid M (2015). Characterisation of the subaquatic groundwater discharge that maintains the permanent stratification within Lake Kivu; East Africa. PLoS ONE.

[11] Llirós M (2015). Pelagic photoferrotrophy and iron cycling in a modern ferruginous basin. Sci. Rep..

[12] Camacho A, Walter XA, Picazo A, Zopfi J (2017). Photoferrotrophy: remains of an ancient photosynthesis in modern environments. Front. Microbiol..

[13] Planavsky NJ (2011). Widespread iron-rich conditions in the mid-Proterozoic ocean. Nature.

[14] Morana C (2016). Chemoautotrophy and anoxygenic photosynthesis within the water column of a large meromictic tropical lake (Lake Kivu, East Africa). Limnol. Oceanogr..

[15] Roland, F. A. E. *Biogeochemical Processing of Greenhouse Gases (Methane and Nitrous Oxide) in Meromictic Lakes*. Ph.D thesis, University of Liège, (2017).

[16] Weiss RF (1981). Determinations of carbon dioxide and methane by dual catalyst flame ionization chromatography and nitrous oxide by electron capture chromatography. J. Chromatogr. Sci..

[17] APHA. *Standard Methods for the Examination of Water and Wastewater*. Vol. 2 (American Public Health Association, Washington DC, 1998).

[18] Miranda KM, Espey MG, Wink DA (2001). A rapid, simple spectrophotometric method for simultaneous detection of nitrate and nitrite. Nitric Oxide-Biol. Ch..

[19] Westwood, D. in *Methods for the Examination of Waters and Associated Materials* (ed HMSO) (Stationery Office Books, London, United Kingdom, 1981).

[20] Cline JD (1969). Spectrophotometric determination of hydrogen sulfide in natural waters. Limnol. Oceanogr..

[21] Roland FAE (2018). Anaerobic methane oxidation and aerobic methane production in an east African great lake (Lake Kivu). J. Great Lakes Res..

[22] Pasche N (2009). Physical and biogeochemical limits to internal nutrient loading of meromictic lake kivu. Limnol. Oceanogr..

[23] Raghoebarsing AA (2006). A microbial consortium couples anaerobic methane oxidation to denitrification. Nature.

[24] Sarmento H, Isumbisho M, Descy J-P (2006). Phytoplankton ecology of Lake Kivu (eastern Africa). J. Plankton Res..

[25] İnceoğlu Ö (2015). Distribution of bacteria and archaea in meromictic tropical Lake Kivu (Africa). Aquat. Microb. Ecol..

[26] Llirós M, Casamayor EO, Borrego C (2008). High archaeal richness in the water column of a freshwater sulfurous karstic lake along an interannual study. FEMS Microbiol. Ecol..

[27] Shah V (2011). Bacterial and Archaea community present in the Pine Barrens Forest of Long Island, NY: unusually high percentage of ammonia oxidizing bacteria. PLoS ONE.

[28] Pruesse E, Peplies J, Glockner FO (2012). SINA: accurate high-throughput multiple sequence alignment of ribosomal RNA genes. Bioinformatics (Oxford, England).

[29] Ludwig W (2004). ARB: a software environment for sequence data. Nucleic Acids Res..

[30] Milucka J (2015). Methane oxidation coupled to oxygenic photosynthesis in anoxic waters. ISME J..

[31] Canfield, D. E., Erik, K. & Bo, T. in *Advances in Marine Biology* Vol. 48 (eds D.E. Canfield, E. Kristensen, & B. Thamdrup) 313–381 (Academic Press, New York, 2005). 10.1016/S0065-2881(05)48017-7 15797449

[32] Overmann J, Cypionka H, Pfennig N (1992). An extremely low-light adapted phototrophic sulfur bacterium from the Black Sea. L&O.

[33] Michiels CC (2017). Iron-dependent nitrogen cycling in a ferruginous lake and the nutrient status of Proterozoic oceans. Nat. Geosci..

[34] Crowe SA (2017). Draft Genome Sequence of the Pelagic Photoferrotroph Chlorobium phaeoferrooxidans. Genome Announc.

[35] Weber HS, Habicht KS, Thamdrup B (2017). Anaerobic methanotrophic Archaea of the ANME-2d cluster are active in a low-sulfate, iron-rich freshwater sediment. Front. Microbiol..

[36] Ettwig KF (2016). Archaea catalyze iron-dependent anaerobic oxidation of methane. PNAS.

[37] Cai C (2018). A methanotrophic archaeon couples anaerobic oxidation of methane to Fe(III) reduction. ISME J..

[38] Crowe S (2011). The methane cycle in ferruginous Lake Matano. Geobiology.

[39] Riedinger N (2014). An inorganic geochemical argument for coupled anaerobic oxidation of methane and iron reduction in marine sediments. Geobiology.

[40] Sivan O (2011). Geochemical evidence for iron-mediated anaerobic oxidation of methane. Limnol. Oceanogr..

[41] Canfield DE, Thamdrup B, Hansen JW (1993). The anaerobic degradation of organic matter in Danish coastal sediments: iron reduction, manganese reduction, and sulfate reduction. Geochim. Cosmochim. Acta.

[42] Jones C (2011). Biogeochemistry of manganese in ferruginous Lake Matano, Indonesia. Biogeosciences.

[43] Oswald K (2016). Methanotrophy under versatile conditions in the water column of the ferruginous Meromictic Lake La Cruz (Spain). Front. Microbiol..

[44] Lovley DR, Klug MJ (1983). Sulfate reducers can outcompete methanogens at freshwater sulfate concentrations. Appl. Environ. Microbiol..

[45] Roberson AL, Roadt J, Halevy I, Kasting JF (2011). Greenhouse warming by nitrous oxide and methane in the Proterozoic Eon. Geobiology.

[46] Pavlov AA, Hurtgen MT, Kasting JF, Arthur MA (2003). Methane-rich proterozoic atmosphere?. Geology.

[47] Catling DC, Claire MW, Zahnle KJ (2007). Anaerobic methanotrophy and the rise of atmospheric oxygen. Philos. Trans. A Math. Phys. Eng. Sci..

[48] Olson SL, Reinhard CT, Lyons TW (2016). Limited role for methane in the mid-Proterozoic greenhouse. PNAS.

[49] Sauterey B, Charnay B, Affholder A, Mazevet S, Ferrière R (2020). Co-evolution of primitive methane-cycling ecosystems and early Earth’s atmosphere and climate. Nat. Commun..

[50] Canfield DE, Rosing MT, Bjerrum C (2006). Early anaerobic metabolisms. *Philos*. Trans. R. Soc. Lond. B Biol. Sci..

[51] Zahnle K, Claire M, Catling D (2006). The loss of mass-independent fractionation in sulfur due to a Palaeoproterozoic collapse of atmospheric methane. Geobiology.

[52] Papineau D, Mojzsis SJ, Schmitt AK (2007). Multiple sulfur isotopes from Paleoproterozoic Huronian interglacial sediments and the rise of atmospheric oxygen. Earth Planet. Sci. Lett..

[53] Konhauser KO (2009). Oceanic nickel depletion and a methanogen famine before the Great Oxidation Event. Nature.

[54] Bray MS (2017). Shifting microbial communities sustain multiyear iron reduction and methanogenesis in ferruginous sediment incubations. Geobiol..

[55] Haqq-Misra JD, Domagal-Goldman SD, Kasting PJ, Kasting JF (2008). A revised, hazy methane greenhouse for the Archean Earth. Astrobiology.

[56] Pavlov AA, Kasting JF, Brown LL, Rages KA, Freedman R (2000). Greenhouse warming by CH_4_ in the atmosphere of early Earth. J Geophys Res.

[57] David LA, Alm EJ (2010). Rapid evolutionary innovation during an Archaean genetic expansion. Nature.

[58] Kopp RE, Kirschvink JL, Hilburn IA, Nash CZ (2005). The Paleoproterozoic snowball Earth: a climate disaster triggered by the evolution of oxygenic photosynthesis. PNAS.

