# Supplementary material for: The possible occurrence of iron-dependent anaerobic methane oxidation in an Archean Ocean analogue
Source: Sci Rep. 2021 Jan 15;11:1597. doi: 10.1038/s41598-021-81210-x (PMC7810693; doi:10.1038/s41598-021-81210-x)
Supplement: Supplementary file 1 — Supplementary Information. [file 41598_2021_81210_MOESM1_ESM.docx]

**The possible occurrence of iron-dependent anaerobic methane oxidation in an Archean Ocean analogue - Supplemental material**

**Fleur A.E. Roland^1*^, Alberto V. Borges^1^, François Darchambeau^1^, Marc Llirós^2ꜛ^, Jean-Pierre Descy^1^, and Cédric Morana^3^**

^1^ Chemical Oceanography Unit, Université de Liège, Belgium

^2^ Department of Genetics and Microbiology, Universitat Autònoma de Barcelona, Spain

^3^ Department of Earth and Environmental Sciences, Katholieke Universiteit Leuven (KU Leuven), Belgium

*Corresponding author: Fleur A.E. Roland (froland@uliege.be)

ꜛCurrent: Girona Biomedical Research Institute, Salt, Catalunya, Spain

| **Table S1**: Depth (m) where CH_4_ oxidation was observed, presence (+) or absence (-) of oxygen (O_2_), CH_4_ oxi = CH_4_ oxidation rates (µmol L^-1^ d^-1^) calculated based on a linear regression, [CH_4_]_in_ = initial CH_4_ concentrations (µmol L^-1^) from which the linear regression begins, %CH_4_ = percentage of initial CH_4_ consumed, and time (h) required for this consumption (time lapse during which the linear regression was applied to calculate CH_4_ oxidation rates), without and with molybdate added (- Mo and + Mo, respectively), for all field campaigns. -/+ Mo = without and with molybdate. | | | | | | | | |
| --- | --- | --- | --- | --- | --- | --- | --- | --- |
| **Depth**  **(m)** | **O_2_** | **CH_4_ oxi**  **(µmol L^-1^ d^-1^)** | | **[CH_4_]_in_**  **(µmol L^-1^)** | **%CH_4_** | | **Time**  **(h)** | |
|  |  | **-Mo** | **+Mo** | **-/+ Mo** | **-Mo** | **+Mo** | **-Mo** | **+Mo** |
| *May 2013* | | | | | | | | |
| 9 | + | 0.02 ± 0.0 | 0 ± 0 | 0.5 ± 0 | 37 ± 1 | 0 ± 0 | 105 | 75 |
| 9.5 | + | 0.12 ± 0.03 | 0 ± 0 | 0.8 ± 0.1 | 54 ± 8 | 42 ± 11 | 105 | 105 |
| 10 | + | 0.05 | 0 ± 0 | 0.7 | 40 | 34 | 105 | 105 |
| 10.5 | + | 0.0 ± 0.0 | 0 ± 0 | 0.6 ± 0 | 24 ± 0 | 30 ± 0 | 105 | 105 |
| 11 | + | 0 ± 0 | 0 ± 0 | 0.6 ± 0 | 0 ± 0 | 29 ± 9 | 75 | 105 |
| 11.5 | - | 50.9 ± 0.8 | 20.8 ± 1.6 | 139 ± 33 | 45 ± 13 | 31 ± 17 | 75 | 60 |
| 12 | - | 6.3 ± 0.6 | 22.6 ± 0.8 | 200 ± 4 | 16 ± 2 | 10 ± 2 | 105 | 105 |
| 13 | - | 2.2 ± 0.8 | 0.8 ± 0.6 | 209 ± 4 | 4 ± 2 | 3 ± 2 | 105 | 105 |
| *September 2013* | | | | | | | | |
| 11.5 | - | 21.4 ± 2.2 | 20.0 ± 2.2 | 132 ± 11 | 82 ± 2 | 80 ± 2 | 105 | 105 |
| 12 | - | 4.6 ± 1.0 | 7.5 ± 0.7 | 151 ± 5 | 15 ± 3 | 24 ± 3 | 105 | 105 |
| 13 | - | 9.0 ± 0.3 | 33.6 ± 1.1 | 174 ± 1 | 18 ± 1 | 21 ± 1 | 75 | 30 |
| *August 2014* | | | | | | | | |
| 8.75 | + | 0.1 ± 0.1 | 0 ± 0 | 0.4 ± 0.2 | 31 ± 26 | 0 ± 0 | 30 | 75 |
| 9 | + | 0.8 ± 0.4 | 0 ± 0 | 0.6 ± 0.1 | 49 ± 10 | 45 ± 13 | 10 | 10 |
| 9.25 | + | 0.1 ± 0.0 | 0 ± 0 | 0.4 ± 0 | 27 ± 8 | 20 ± 1 | 30 | 15 |
| 10 | + | 0.1 ± 0.0 | 0 ± 0 | 0.5 ± 0 | 30 ± 1 | 26 ± 1 | 25 | 75 |
| 10.25 | + | 0.2 ± 0.0 | 0 ± 0 | 1 ± 0 | 30 ± 1 | 48 ± 1 | 48 | 24 |
| 10.5 | + | 0.1 ± 0.0 | 0 ± 0 | 1 ± 0 | 32 ± 6 | 50 ± 4 | 48 | 48 |
| 10.75 | + | 0.2 ± 0.0 | 0.2 ± 0.1 | 0.9 ± 0.1 | 34 ± 4 | 32 ± 4 | 48 | 10 |
| 11.25 | - | 0.5 ± 0.1 | 0 ± 0 | 2 ± 0 | 47 ± 9 | 55 ± 8 | 48 | 70 |
| 11.5 | - | 8.1 ± 0.6 | 12.8 ± 1.3 | 5 ± 0 | 78 ± 1 | 79 ± 1 | 10 | 5 |
| 11.75 | - | 37.5 | 205.8 | 43 ± 20 | 93 ± 3 | 95 ± 2 | 24 | 5 |
| 12 | - | 48.0 ± 6.9 | 269.0 ± 14.5 | 130 ± 7 | 35 ± 3 | 92 ± 0 | 24 | 12 |

| Table S2: Vertical fluxes (mmol m^-2^ d^-1^) of the different potential electron acceptors for AOM and their reduced forms, for the three field campaigns. Mn^2+^, Fe^2+^, NH_4_^+^ and HS^-^ fluxes were calculated with the extremes values of vertical diffusion and advection coefficients reported in the Material and Methods section. Fe and Mn oxides fluxes were calculated based on the inputs by the rivers* and the Fe^2+^ and Mn^2+^ vertical fluxes. | | | |
| --- | --- | --- | --- |
|  | **May 2013** | **September 2013** | **August 2014** |
| Fe oxides | 6.0 – 25.0 | 7.0 – 34.0 | 9.0 – 49.0 |
| Fe^2+^ | 3.0 – 21.5 | 4.2 – 30.2 | 6.2 – 46.0 |
| Mn oxides | 0.2 – 1.1 | 0.2 | 1.3 – 9.0 |
| Mn^2+^ | 0.1 – 1.0 | 0.1 | 1.2 – 8.9 |
| NH_4_^+^ | N.d. | 0.6 – 4.3 | 1.3 – 9.5 |
| HS^-^ | 0 | 0.01 – 0.1 | 0.01 – 0.05 |

N.d., not determined

* The inputs by the rivers were calculated based upon the annual inputs of total Fe and Mn in the 5 main rivers entering in Kabuno Bay, and the annual discharge of these rivers, according to the following equations:

1) Inputs = ((D * C)/365)/1000

where inputs are the Fe or Mn inputs in Kabuno Bay from each river (mol d^-1^), D is the discharge (m³ yr^-1^), and C is the annual Fe or Mn concentrations (mmol m^-3^),

2) Total inputs = Σ Inputs/A*1000

where total inputs (mmol m^-2^ d^-1^) are the inputs from the five main rivers, and A is the area of Kabuno Bay (m²).

The inputs by the rivers were then estimated to 3.28 and 0.1 mmol m^-2^ d^-1^ for Fe and Mn oxides, respectively.

**Table S3**. Sequence similarity values (%) among 16S rRNA gene sequences from the most abundant AAA OTUs (0.03 cut-off value) with respect to reference genomes within Candidatus *Methanoperedens* archaeal group.

|  | Cand. *Methanoperedens sp. BLZ1*  (LKCM01000080) | *Kabuno Bay OTU001* | Cand. *Methanoperedens nitroreducens*  (JMIY01000002) | *Kabuno Bay OTU005* |
| --- | --- | --- | --- | --- |
| Cand. *Methanoperedens sp. BLZ1*  (LKCM01000080) | 100.0 | 98.6 | 95.5 | 94.6 |
| *Kabuno Bay OTU001* | 98.6 | 100.0 | 94.6 | 94.1 |
| Cand. *Methanoperedens nitroreducens*  (JMIY01000002) | 95.5 | 94.6 | 100.0 | 93.7 |
| *Kabuno Bay OTU005* | 94.6 | 94.1 | 93.7 | 100.0 |

**Supplementary discussion: comparison of AOM rates reported in Kabuno Bay with other meromictic lakes**

We showed that CH_4_ oxidation in Kabuno Bay was solely anaerobic, what can be mainly explained by the vertical structure of the water column. In the main basin of Lake Kivu, the proportion of the aerobic CH_4_ oxidation is much higher (Table S4). The water column of the main basin is indeed characterized by a thicker oxic layer and, contrary to Kabuno Bay, is strongly influenced by the season. The stratification deepens during the dry season, allowing a higher input of CH_4_ from anoxic waters to oxic waters by turbulent mixing, and thus higher aerobic CH_4_ oxidation rates^1^.

AOM rates measured in this study were 2 to 1250 times higher in Kabuno Bay than in many other meromictic lakes (Table S4). To our best knowledge, only Lake Matano showed higher AOM rates, but if we normalize AOM rates with the CH_4_ concentrations, microbial communities in Kabuno Bay are capable to consume, per day, on average 7-36 % of the CH_4_ present, while in the main basin of Lake Kivu and in Lake Matano, they are able to consume on average 0.3-2 % and 24-35 %, respectively. The relatively fast CH_4_ turnover observed in our study might reflect the higher availability of electron acceptors needed to fuel AOM due to the sharpness of the chemocline in Kabuno Bay. In the main basin of Lake Kivu, the imbalance between the measured CH_4_ oxidation rates and vertical substrate fluxes did not allow to clearly identify the main electron acceptor that fuels AOM, but instead suggests that a substantive amount of inorganic molecules is effectively recycled in the chemocline^1^.

| **Table S4:** Methane oxidation rates in other meromictic lakes. Ferruginous lakes are highlighted in grey. | | |
| --- | --- | --- |
| **Lake** | **Aerobic CH_4_ oxidation**  **(µmol L^-1^ d^-1^)**  **(CH_4_ concentrations;**  **µmol L^-1^)** | **AOM**  **(µmol L^-1^ d^-1^)**  **(CH_4_ concentrations;**  **µmol L^-1^)** |
| Kabuno Bay (East Africa)^*^ | 0.03 – 0.14 (0.5 – 0.8) | 0.1 – 51 (1 – 140) |
| Pavin (France)^2^ | 0.001 – 0.05 (0.06 – 0.35) | 0.4 (285 – 785) |
| La Cruz (Spain)^3^ | 0.7 – 1.8 (<5) | 2.5 – 2.8 (<100) |
| Matano (Indonesia)^4^ | 0.0004 – 0.003 (0.5) | 4.2 – 117 (12 – 484) |
| Kivu (East Africa)^1^ | 0.02 – 27 (0.2 – 42) | 0.2 – 16 (65 – 689) |
| Big Soda (US)^5^ | 0.0013 (0.1) | 0.06 (50) |
| Marn (Sweden)^6^ | 0.8 (10) | 2.2 (55) |
| Tanganyika^7^ | 0.1-0.96 (<10) | 0.24-1.8 (~10) |

^*^, present work

|  Western  Basin  Northern  Basin  Southern  Basin  Bukavu Bay |
| --- |
| **Figure S1**: Map of Lake Kivu, showing the different basins and bays, and focus on Kabuno Bay with the sampling site (black plot). Created with ESRI ArcGIS products (version 10.7), with data from Hydrosheds^8^. |

|  |
| --- |
| **Figure S2**: Specifically designed sampler for the sampling of the water column of Kabuno Bay. |

|  |
| --- |
| **Figure S3**: Vertical profiles of (a-d) the physico-chemical parameters and (e) the CH_4_ concentrations (µmol L^-1^) during the three field campaigns (blue: May 2013; green: September 2013; orange: August 2014) |

|  |
| --- |
| **Figure S4**: Vertical profiles of Chlorophyll a concentrations (µg L^-1^) in May 2013 (orange), September 2013 (green) and August 2014 (blue) |

**References**

1. Roland, F.A.E. et al. Anaerobic methane oxidation and aerobic methane production in an east African great lake (Lake Kivu)*.* *J. Great Lakes Res.,* **44**, 1183-1193 (2018).

2. Lopes, F. et al. Biogeochemical modelling of anaerobic vs. aerobic methane oxidation in a meromictic crater lake (Lake Pavin, France)*.* *Appl. Geochem.,* **26**, 1919-1932 (2011).

3. Oswald, K. et al. Methanotrophy under Versatile Conditions in the Water Column of the Ferruginous Meromictic Lake La Cruz (Spain)*.* *Front. Microbiol.,* **7**, (2016).

4. Sturm, A. et al. Rates and pathways of CH_4_ oxidation in ferruginous Lake Matano, Indonesia*.* *Biogeosciences Discuss.,* **2016**, 1-34 (2016).

5. Iversen, N., Oremland, R.S. & Klug, M.J. Big Soda Lake (Nevada). 3. Pelagic methanogenesis and anaerobic methane oxidation*.* *Limnol. Oceanogr.,* **32**, 804-814 (1987).

6. Bastviken, D., Ejlertsson, J. & Tranvik, L. Measurement of methane oxidation in lakes: A comparison of methods*.* *Environ. Sci. Technol.,* **36**, 3354-3361 (2002).

7. Rudd, J.W. Methane oxidation in Lake Tanganyika (East Africa)*.* *Limnol. Oceanogr.,* **25**, 958-963 (1980).

8. Lehner, B., Verdin, K. & Jarvis, A. New global hydrography derived from spaceborne elevation data*.* *Eos, Transactions, AGU,* **89**, 93-94 (2008).
